# Primary Systemic Amyloidosis: A Case Report

**DOI:** 10.31729/jnma.8297

**Published:** 2023-10-31

**Authors:** Saurav Sen Oli, Abhishek Jha, Anisha Karki, Shova Sapkota, Laxman Adhikari

**Affiliations:** 1 Kathmandu Medical College and Teaching Hospital, Sinamangal, Kathmandu, Nepal; 2 Department of Nephrology, Kathmandu Medical College and Teaching Hospital, Sinamangal, Kathmandu, Nepal

**Keywords:** *cardiomyopathies*, *case reports*, *primary amyloidosis*

## Abstract

Primary systemic amyloidosis is a systemic disease characterised by the deposition of misfolded proteins extracellularly in different organs without any known cause in the background, eventually leading to multiorgan dysfunction and death. The incidence of primary amyloidosis is estimated at 5.1-12.8 cases per million, with a poor prognosis. We report a case of a 69-year male with lower back pain, shortness of breath, and anasarca diagnosed as primary systemic amyloidosis by serumfree light chain assay and kidney needle biopsy. He was started on intravenous bortezomib and dexamethasone. Though he adhered to his medications, with time he developed renal insufficiency marked by azotemia following which hemodialysis was performed. Primary systemic amyloidosis is a rare clinical condition with a very poor prognosis. Further studies are needed to understand the proper pathophysiology and treatment of the disease.

## INTRODUCTION

Amyloidosis is a condition characterised by deposition of misfolded proteins within tissues and organs.^1^ Primary amyloidosis also referred to as light chain (AL) amyloidosis has the deposition of monoclonal immunoglobulin (Ig) light chains in organs like the heart, kidney, liver, gastrointestinal (GI) tract, peripheral and autonomic nervous system.^2,3^ It is a rare condition with an incidence of 5.1-12.8 per million per year in the USA.^4^ We hereby present a case of primary amyloidosis presented with chronic kidney disease and heart failure which emphasize early diagnosis and intervention in similar cases.

## CASE REPORT

A 69-year-old male presented with complaints of generalized body swelling for one and a half months, pitting type, initially on bilateral lower limbs progressively ascending to the thigh, scrotal area and then abdomen; decreased urine output for 15 days. On clinical examination, his blood pressure was 100/60 mm Hg and oxygen saturation of 83% in room air, bilateral pitting oedema was present over lower limbs and trunk. On respiratory system examination, bilateral basal crepitations were present. Arterial blood gas analysis revealed metabolic acidosis. Laboratory studies showed Hb 9.5 gm/dl, blood urea 213 mg/dl and serum creatinine 3.5 mg/dl and normal complete blood counts. His intact Parathyroid hormone level was increased. His echocardiography and N-terminal pro-brain natriuretic peptide (NT Pro BNP) of 787 suggested severe left ventricular systolic dysfunction with an ejection fraction of 20-25%, severe concentric left ventricular hypertrophy, severe tricuspid regurgitation with moderate pulmonary arterial hypertension and grade III diastolic dysfunction. His chest x-ray and high-resolution computed tomography (HRCT) showed centrilobular nodular opacities with surrounding ground glass opacities diffusely in the bilateral lung field with moderate right and minimal left-sided pleural effusion.

Our patient is a known case of hypertension for 20 years and is under medication. Ten years back, he started having lower back pain insidious in onset, dull aching, and non-radiating in nature and magnetic resonance imaging (MRI) of the spine revealed disc desiccation at multiple levels with osteophytes suggestive of degenerative changes. He also had albuminuria in his urine through routine microscopic examination. His bone marrow biopsy and multiple myeloma panel test were done with the suspicion of multiple myeloma. Bone marrow aspirate smears showed <5% plasma cells and bone marrow biopsy revealed normal marrow elements with normal maturation, two foci blood vessels showing thickened walls with amorphous penile material and no obvious increase in plasma cells. His serum protein electrophoresis had shown monoclonal gammopathy as, monoclonal spike (M spike) 1.44 g/dl; increased beta-2 microglobulin 3846 ng/ml; in free light chain assay kappa and lambda were increased with normal kappa/lambda ratio as 28.50 mg/l, 173 mg/l and 0.16 respectively; urine protein electrophoresis was negative for bence jones protein but showed M spike.

Renal biopsy showed renal amyloidosis with focal glomerular and vascular deposition of amyloid, lambda light chain dominance along areas of glomerular and extraglomerular amyloid deposition in direct immunofluorescence. Echocardiography revealed concentric left ventricular hypertrophy likely infiltrates with amyloid, severe left ventricle systolic dysfunction with an ejection fraction of 20-25% and left ventricular diastolic dysfunction grade III. Abdominal fat pad biopsy was negative for congo red staining and did not show any polarization. Based on these findings, this case was diagnosed as primary systemic amyloidosis with renal and cardiac stage III. The patient was then started on bortezomib and dexamethasone which were stopped after haematological remission but later again started as bortezomib, dexamethasone and pomalidomide regimen.

At the time of presentation to our centre, he had features suggestive of fluid overload, a possible chest infection and heart failure; investigations also showed metabolic acidosis along with raised blood urea, and serum creatinine. He was admitted to the intensive care unit (ICU) and was started on diuretics, prophylactic antibiotics, oxygen via nasal prongs and haemodialysis planned. He underwent 7 sessions of hemodialysis via subclavian-jugular vascular access with heparin as an anticoagulant at the rate of 3 sessions a week, each session ranging from 2 to 4 hr. The patient was transfused with a total of 3 pints of packed red cells over the course of hemodialysis. IV noradrenaline was administered as cardiac support for low blood pressure on tapering dose. At the time of discharge, the patient was symptomatically better, his oedema had subsided, laboratory values were within normal limits, vitals were stable and he was passing urine and stool on a regular basis. The patient was advised for a regular follow-up, renal diet and next dialysis to be planned on the basis of renal function test reports in the subsequent visits.

## DISCUSSION

Primary amyloidosis is a rare condition with male to female ratio of 3:2 and peak incidence in the 7^th^ to 8^th^ decade of life.^5^ 10-15% of patients with primary amyloidosis are found to also have associated multiple myeloma.^6^ Primary amyloidosis is usually seen in asymptomatic or smouldering multiple myeloma but is unusual in patients with symptomatic multiple myeloma, non-Hodgkin's lymphoma, Waldenstrom macroglobulinemia and another beta lymphoproliferative disorder.^2,3^ In our case, the patient is a 69-year-old male who developed primary systemic amyloidosis with no diagnosed disease in the background.

Primary amyloidosis affects multiple organs in the body except the central nervous system. The heart and kidney are the most commonly involved organs though cases of liver, GI tract, tongue, skin, soft tissues, and peripheral and autonomic nervous system involvement have also been reported. Patients usually present with fatigue, weight loss and features of multiple system involvement like peripheral oedema, ascites, neuropathy, dyspnea, diarrhoea, macroglossia, myopathy, coagulopathy etc.^3,5^ Our patient presented with a history of generalised body swelling and decreased urine output.

In the case of renal amyloidosis, the disease might progress in the sequence of microscopic hematuria, subnephrotic proteinuria to nephrotic range proteinuria, and acute kidney injury which ultimately might lead to chronic kidney disease if not diagnosed and treated on time. Among all symptoms, proteinuria is the most common presentation while only 15-30% of patients with renal symptoms develop end-stage renal disease.^5,7^ In our case, the patient had a nephrotic range of proteinuria, generalised body swelling, raised blood urea/creatinine, decreased urine output, increased intact parathyroid hormone, and anaemia suggesting chronic kidney disease requiring renal replacement therapy.

Cardiac amyloidosis is associated with the worst prognosis and results from amyloid deposition in the myocardium. It is characterised by infiltrative cardiomyopathy which ultimately results in heart failure.^2,8^ A case of relapsing decompensated heart failure with severe concentric hypertrophy and diastolic dysfunction was reported which is similar to our case where our patient has concentric left ventricular hypertrophy, and diastolic dysfunction with a reduced ejection fraction of 20-25%.^8^

Diagnosis of primary amyloidosis is difficult and hence requires a high degree of suspicion based on clinical features.^5^ Serum protein and urine protein electrophoresis, immunofixation electrophoresis and serum free light chain assay can be used as screening tests for diagnosis of primary amyloidosis and has a sensitivity of 99% if all the tests are combined.^3^ Diagnosis is confirmed only with biopsy, less invasive procedures such as abdominal fat pad aspiration should be done prior to biopsy of internal organs. Amyloid deposits are stained with congo red and develop apple-green birefringence under polarised light.^2^ In our case, serum and urinary protein electrophoresis, free serum light chain assay, and immunoturbidimetry were done first followed by an abdominal fat pad, and renal and bone marrow biopsy for confirmation of diagnosis.

Chemotherapy and autologous stem-cell transplantation (SCT) are the mainstays of treatment for systemic amyloidosis.^1^ According to the phase 3 ANDROMEDA trial, which was recently published, treatment with daratumumab, cyclophosphamide, bortezomib, and dexamethasone is becoming the standard of care for patients with systemic primary amyloidosis.^9^ Bortezomib is the preferred treatment in patients under renal replacement therapy as it does not require dose adjustment.^10^

Our patient received a combination of bortezomib and dexamethasone followed by the addition of immunomodulator pomalidomide. Patients presenting with clinical features in the spectrum of renal impairment should be suspected of primary amyloidosis. Early evaluation and diagnosis can prevent the deterioration of disease to chronic renal failure with timely interventions.

## References

[1] Cuddy SAM, Falk RH (2020). Amyloidosis as a systemic disease in context.. Can J Cardiol..

[2] Reimann HA, Sahyoun PF, Chaglassian HT (1954). Primary amyloidosis; relationship to secondary amyloidosis and report of a case.. AMA Arch Intern Med..

[3] Baker KR, Rice L (2012). The amyloidoses: clinical features, diagnosis and treatment.. Methodist Debakey Cardiovasc J..

[4] Falk RH, Comenzo RL, Skinner M (1997). The systemic amyloidoses.. N Engl J Med..

[5] Jensen CE, Byku M, Hladik GA, Jain K, Traub RE, Tuchman SA (2022). Supportive care and symptom management for patients with immunoglobulin light chain (al) amyloidosis.. Front Oncol.

[6] Hu H, Huang D, Ji M, Zhang S (2020). Multiple myeloma with primary amyloidosis presenting with digestive symptoms: a case report and literature review.. Arab J Gastroenterol..

[7] Hogan JJ, Alexander MP, Leung N (2019). Dysproteinemia and the kidney: core curriculum 2019.. Am J Kidney Dis..

[8] Cappannoli L, Ciliberti G, Restivo A, Palumbo P, D'Alo F, Sanna T (2022). 'Here comes the story of the hurricane': a case report of AL cardiac amyloidosis and myocardial bridging.. Eur Heart J Case Rep.

[9] Kawano Y, Hata H, Takashio S, Tsujita K, Ueda M, Matsuoka M (2022). Daratumumab, lenalidomide and dexamethasone in newly diagnosed systemic light chain amyloidosis patients associated with multiple myeloma.. Br J Haematol..

[10] Renal Impairment of Multiple Myeloma Collaborative Study Group. (2017). [Expert consensus for the diagnosis and treatment of patients with renal impairment of multiple myeloma].. Zhonghua Nei Ke Za Zhi..

